# Identification and Validation of Cytotoxicity-Related Features to Predict Prognostic and Immunotherapy Response in Patients with Clear Cell Renal Cell Carcinoma

**DOI:** 10.1155/2024/3468209

**Published:** 2024-08-30

**Authors:** Junxiao Yu, Bowen Zhao, You Yu

**Affiliations:** ^1^ Department of Urology The First Affiliated Hospital of Harbin Medical University, Harbin 150010, China; ^2^ Department of Oral and Maxillofacial Surgery The First Affliated Hospital of Harbin Medical University, Harbin 150010, China; ^3^ Department of Newborn Surgery The Sixth Affiliated Hospital of Harbin Medical University, Harbin 150023, China

## Abstract

**Background:**

Clear cell renal cell carcinoma (ccRCC) is a renal cortical malignancy with a complex pathogenesis. Identifying ideal biomarkers to establish more accurate promising prognostic models is crucial for the survival of kidney cancer patients.

**Methods:**

Seurat R package was used for single-cell RNA-sequencing (scRNA-seq) data filtering, dimensionality reduction, clustering, and differentially expressed genes analysis. Gene coexpression network analysis (WGCNA) was performed to identify the cytotoxicity-related module. The independent cytotoxicity-related risk model was established by the survival R package, and Kaplan–Meier (KM) survival analysis and timeROC with area under the curve (AUC) were employed to confirm the prognosis and effectiveness of the risk model. The risk and prognosis in patients suffering from ccRCC were predicted by establishing a nomogram. A comparison of the level of immune infiltration in different risk groups and subtypes using the CIBERSORT, MCP-counter, and TIMER methods, as well as assessment of drug sensitivity to conventional chemotherapeutic agents in risk groups using the pRRophetic package, was made.

**Results:**

Eleven ccRCC subpopulations were identified by single-cell sequencing data from the GSE224630 dataset. The identified cytotoxicity-related T-cell cluster and module genes defined three cytotoxicity-related molecular subtypes. Six key genes (SOWAHB, SLC16A12, IL20RB, SLC12A8, PLG, and HHLA2) affecting prognosis risk genes were selected for developing a risk model. A nomogram containing the RiskScore and stage revealed that the RiskScore contributed the most and exhibited excellent predicted performance for prognosis in the calibration plots and decision curve analysis (DCA). Notably, high-risk patients with ccRCC demonstrate a poorer prognosis with higher immune infiltration characteristics and TIDE scores, whereas low-risk patients are more likely to benefit from immunotherapy.

**Conclusions:**

A ccRCC survival prognostic model was produced based on the cytotoxicity-related signature, which had important clinical significance and may provide guidance for ccRCC treatment.

## 1. Introduction

Renal cancer is a frequent cancer of the urinary system accounting for about 2-3% of adult malignancies [1]. Clear cell renal cell carcinoma (ccRCC) is a rather prevalent and aggressive histological subtype representing about 80–90% of metastatic renal cancer cases [2]. Patients with ccRCC typically have symptoms such as flank pain, abdominal masses, and hematuria, but most ccRCC patients are asymptomatic at the early stage, so about 1/3 of patients have distant metastases at diagnosis and about 1/4 will experience relapse and metastases after surgery, making to a low overall survival and poor prognosis for ccRCC patients [3, 4]. Currently, a substantial proportion of patients are insensitive to chemotherapy and conventional radiotherapy and surgical intervention is still the main treatment for ccRCC patients in the early stage [5]. However, about 25% of patients will recur or develop tumor metastasis after surgery. In other words, surgery cannot completely address the treatment needs of these ccRCC patients [6, 7]. Currently, there are studies confirming that immunotherapeutic approaches with different targets are effective in improving the survival rate of cancer patients [8, 9]. Among these, immune checkpoint blockade is a novel immunotherapy that reduces inhibitory signaling and restores tumor-specific T-cell-mediated immune responses [10]. Several anti-PD-1/PD-L1 drugs have been approved for the treatment of advanced renal cell carcinoma and have shown acceptable efficacy [11]. However, the effectiveness of the treatment is limited due to the complex tumor microenvironment (TME), and investigations on pathogenesis and prognostic predictors of ccRCC including new effective immunotherapeutic targets for improved clinical outcomes are still imperative.

Immune cells in TME exert a vital role in tumor progression regulation and could be used as a therapeutic target [12]. The characteristics of TME strongly influence the response of ccRCC patients to immunotherapy because the tumor is prone to immune infiltration [13]. The clinical success of antitumor immune response involved the activation and synergistic action of multiple tumor-infiltrating lymphocytes [14]. T cells as a type of tumor-infiltrating cells are closely related to the immunosuppressive properties of ccRCC [15]. Recent studies based on various biological aspects of ccRCC have revealed features associated with immune infiltration. Specific lymphocyte-related characteristics such as CD8+ T cells [16], TNFRSF9 + CD8+ T cells [17], and the CXCL13+ CD8+ T cells [18] have been found in ccRCC. The CD8+ T cells are cytotoxic T lymphocytes, which are key players in eliminating the pathogen-infected cells or tumorigenic via the secreted cytotoxic proteins (perforin and granzymes) [19, 20]. In general, T-cell exhaustion represents an immune-functionally impaired status converted formation antitumor status of CD8+ in the TME, which is considered to be one of the main factors contributing to the low response rate to immunotherapy [21]. Therefore, immunotherapy targeting the conversion of exhausted T cells recovering to an activated state in ccRCC has recently received great research attention. The infiltration of CD8+ T cells in the TME of ccRCC presents highly heterogeneous phenotypes, which are closely associated with the immunotherapy response [22]. High levels of immune-evasive biomarkers and enhanced immunosuppressive infiltrations are often associated with a poor prognosis in patients with ccRCC [23, 24]. However, interrelated association between the effectiveness of immunotherapy and the degree of CD8+ T-cell infiltration in ccRCC is still not clear [25, 26]. Therefore, additional analysis of biomarkers linked to CD8+ T cells is urgently required in order to find novel prognostic markers that will guide immunotherapy for ccRCC.

This study characterized CD8+ T-cell-associated molecule clusters by the cytotoxic score in ccRCC; the WGCNA was used to distinguish the modules related to the cytotoxic score in the TCGA dataset. Subsequently, three distinct cytotoxicity-related molecular clusters with different prognoses and clinical characteristics were categorized by unsupervised cluster analysis. Finally, a risk model consisting of six cytotoxicity-related genes that affect prognosis was developed to guide prognosis and provide new insights for personalized immunotherapy.

## 2. Materials and Methods

### 2.1. Data Collection and Processing

Gene Expression Omnibus (GEO) database (https://www.ncbi.nlm.nih.gov/geo/) is a public database that provides the ccRCC single-cell dataset GSE224630, which included six tumor samples. We used the tool of The Cancer Genome Atlas (TCGA) GDC API to download the RNA-sequencing data and clinical grades on Kidney Renal Clear Cell Carcinoma (KIRC, https://cancergenome.nih.gov/) for the training set, which contained a total of 72 normal samples and 530 primary tumor samples (513 tumor samples had complete survival time). The gene expression profiles of RECA-EU/Renal cell carcinoma as a validation set were acquired from the database of the International Cancer Genome Consortium (ICGC), including 91 primary tumor samples.

### 2.2. The Analysis of scRNA Data and Identification of Cytotoxicity-Related Cluster

The single-cell data filtering of the dataset GSE224630 was performed under the criteria that each cell expressed at least 200 genes and each gene is expressed in at least three cells. The proportion of mitochondria and rRNA was calculated by the PercentageFeatureSet function, ensuring that of the 100 <mRNA of each cell <5000, the mitochondrial content is <15%. The samples were normalized by log-normalization, and the highly variable genes were identified by the FindVariableFeatures function based on the variance-stabilization transformations (selection.method = “vst”). The FindIntegrationAnchors function (reduction = “cca”) was used to remove the six samples' batch effect, and the data were consolidated using the IntegrateData method. The FindNeighbors and FindClusters functions were applied for cell unsupervised clustering. In addition, we collected the cell markers from the CellMarker2.0 website, and the cells were reclassified according to the expression of these marker genes. tSNE dimensionality reduction of cells was performed by using the RunTSNE method. Then, we screened these marker genes in different species cells using FindAllMarkers (setting by |log(fold change, FC)| = 0.5, *p* value < 0.05, and min.pct = 0.25). The clusterProfiler was used for Kyoto Encyclopedia of Genes and Genomes (KEGG) enrichment analysis. The above-used functions are in the Seurat package [27]. A signature gene set of T cells was obtained from the previous article [28], and the AUCell R package was used to calculate the cytotoxic score of each T cell [29]. Moreover, the cytotoxic scores of TCGA samples were evaluated by the method of single-sample Gene Set Enrichment Analysis (ssGSEA) through the GSVA package [30], and the samples were grouped based on the median cytotoxic score.

### 2.3. The WGCNA for the Cytotoxicity-Related Module Genes

The limma package in R (|log2(FC)| > 1 and FRD < 0.05) [31] was employed to screen differentially expressed genes (DEGs) in two types of TCGA samples. The gene modules the most associated with the cytotoxic score were identified by WGCNA. After excluding the top 50% of genes with the smallest medium absolute deviation (MAD) in the gene expression profile, the function of pickSoftThreshold in the WGCNA package was used to determine the soft threshold *β* [32], and the gene modules were identified by hierarchical clustering (the minimum module size was at least 50 genes (minModuleSize = 50) and modules were merged if the distance is <0.2). The correlation between modules and clinical features was evaluated by the Spearman method.

### 2.4. Definition of the Cytotoxicity-Related Molecular Subtypes

The intersection between the C0 cluster of T cells, magenta module, and DEGs of ccRCC resulted in 18 overlapping genes. According to the expression of these overlapping genes, a consensus matrix of the expression profile data of TCGA-KIRC was created using the ConsensusClusterPlus R package for sample classification [33]. The “pam” acts as the clustering algorithm, “pearson” acts as the metric distance, and 80% of patients of TCGA-KIRC were incorporated into each bootstrap within 500 bootstraps. Cumulative distribution function (CDF) curves between the number of clustering from 2 to 10 were used to determine the optimal number of clustering and cytotoxicity-related molecular subtypes.

### 2.5. Immune Landscape Analysis of Cytotoxicity-Related Molecular Subtypes

Based on the optimal number of clustering, the TCGA samples were divided into different subtypes. The KM survival analysis [34] was conducted, and subsequently, the survival state of different subtypes was determined. The CIBERSORT algorithm in the estimate R package [35] was used to calculate the relative abundance of 22 types of immune cells. The MCP-counter function of the MCP-counter R package [36] and the TIMER method [37] were used to evaluate the relative proportion of 10 immune cells and the six immune cell scores, respectively. The ESTIMATE algorithm was used to evaluate the immune cell infiltration, including the immune score, stromal score, and ESRIMATEScore, which are positively correlated with immunity, stroma, and the sum of immunity + stroma, respectively [38]. To study the pathways of biological processes, a GSEA (https://software.broadinstitute.org/gsea/index.jsp) was performed using the gene set (h.all.v7.5.1.symbols.gmt, a false discovery rate (FDR) < 0.05) in the MSigDB database through the clusterProfiler R package. At the same time, this gene set was used to calculate the pathway ssGSEA score of different risk groups using the GSVA R package.

### 2.6. Screening of Cytotoxicity-Related Hub Genes and Establishment of RiskScore Model

In order to establish a RiskScore model, the genes with significant prognosis were selected by comparing the differently expressed genes (C1 vs (C2, C3), C2 vs (C1, C3), C3 vs (C1, C2)) among three molecular subtypes using the limma package (setting FDR <0.05 and |log2(FC)| > 1). The coxph function of the survival R package [39] was used in the univariate Cox regression analysis to filter key prognostic genes with *p* < 0.05, followed by applying the least absolute shrinkage and selection operator (Lasso) Cox regression analysis in the glmnet function of the R package to reduce the total gene number [39]. The multivariate Cox regression analysis with the stepwise regression method was performed to determine the final risk factor. After that, a cytotoxicity-related scoring system for ccRCC patients was established by the multivariate Cox results and the expression of genes: cytotoxicity-related RiskScore=Σ*β*i∗Expi (i represents the expression of a risk gene, and *β* is the Cox regression coefficient of the gene). The RiskScore of each patient and the optimal cutoff were calculated by the survminer package [40] for patient's risk classification.

### 2.7. Evaluation of Independent Predictors and Construction of Nomogram on RiskScore

Independent prognostic factors were determined by performing univariate and multivariate Cox regression analysis on the RiskScore and other clinical features, such as stage, age, gender, and grade. The variables with *p* < 0.05 in the univariate and multivariate Cox regression were used to build a nomogram for predicting ccRCC prognosis using the rms R package [41]. The receiver operating characteristic analysis of 1, 3, 5 years with the AUC was applied to identify the classification efficiency of the model using the timeROC R package [42]. The calibration curve was used to assess the predictive accuracy of nomogram model at 1, 3, and 5 years. The decision curve analysis (DCA) was performed to evaluate the reliability of the nomogram.

### 2.8. Immunotherapy Evaluation of Risk Groups

To elucidate the immunotherapy difference in different risk groups, the gene expression of the immune checkpoint and Tumor Immune Dysfunction and Exclusion (TIDE, https://tide.dfci.harvard.edu/) score were analyzed [1]. The TIDE score reflected that the tumor inhibits the function and infiltration of cytotoxic T lymphocytes to achieve immune escape [43], and a high TIME score is not beneficial for immunotherapy.

### 2.9. Drug Sensitivity of Risk Groups

Considering that there exists a prognostic difference in different risk groups, we performed further analysis of conventional drug sensitivity. The lower 50% inhibiting concentration (IC50) value represented enhanced susceptibility to drugs. Utilizing the pRRophetic R package [44] and the pharmacogenomic data of the Genomics of Drug Sensitivity in Cancer (GDSC, https://www.cancerrxgene.org/) [45], we calculated the drug sensitivity in risk groups.

### 2.10. Pathway and Mutation Characteristic Analysis of Risk Groups

The ssGSEA score of each sample in the HALLMARK pathway and the correlation between the RiskScore and ssGSEA score were calculated. Moreover, the molecular characteristics of TCGA-KIRC in the previous pan-cancer study [46] and the mutect2-processed TCGA mutation dataset were used for mutation characteristic analysis. The Fisher test was used to screen genes showing significant high-frequency mutations in different groups.

### 2.11. Statistical Analyses

All the statistical analyses and figures were produced in the R environment (version 3.6.3). The Two-tailed Wilcoxon rank sum test was applied to calculate the differences between two sets of continuous variables. The Pearson or Spearman correlation was used for calculating the correlation matrices. The survival differences were depicted using KM curves with a log-rank test. The Fisher test was used to screen the significant high-frequency mutation genes. Sangerbox (https://sangerbox.com/home.html), which is an interaction-friendly bioinformatics analysis platform, offered analysis support in this paper. A *p* value <0.05 was considered statistically significant.

## 3. Results

### 3.1. Single-Cell Dimension Reduction and Identification of Cytotoxicity-Related Cluster

**Figure 1:**
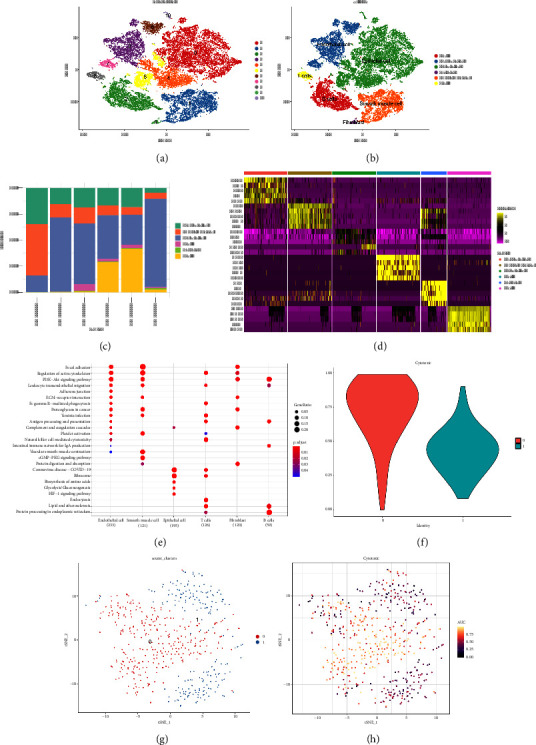
Single-cell analysis and cytotoxicity-related T-cell clustering. (a) The tSNE plot of 11 subgroups' distribution after clustering. (b) tSNE diagram of six species cells' distribution after annotation. (c) The proportion of six species cells in different samples. (d) The heatmap of top five marker genes of six species cells. (e) The KEGG enrichment results of the marker gene in six subgroups. (f) The difference of cytotoxic scores in T-cell subsets. (g) The tSNE plot of two T-cell subsets. (h) The AUC values of T cells with different cytotoxic scores.

We conducted a single-cell analysis of six tumor samples from GSE224630. A total of 26906 cells were identified (Figures S1A–S1C), and these cell samples were further divided into 11 subgroups (clusters 0–10) by dimensionality reduction cluster analysis (Figure 1(a)). According to the expression of 14 marker genes taken from the Cellmarker 2.0, the cells were reclassified into six cell types, including the fibroblasts, T cells, epithelial cells, endothelial cells, smooth muscle cells, and B cells (Figure 1(b)). Among them, epithelial cells, smooth muscle cells, and endothelial cells accounted for the largest proportion, while the T cells had the smallest proportion in the six samples (Figure 1(c)). The expressions of the top five differential marker genes with the most outstanding contributions in T cells are NKG7, CCL4, CCL5, GNLY, and KLRB1 (Figure 1(d)). The KEGG enrichment analysis of these marker genes revealed that the pathway of regulation of actin cytoskeleton, Yersinia infection, Fc gamma R-mediated phagocytosis, endocytosis, and natural killer cell-mediated cytotoxicity were enriched in T cells (Figure 1(e)). The T cells were further divided into 2 T cell subsets by FindClusters (resolution = 0.2), and the C0 cluster had a higher cytotoxic score than the C1 cluster (Figures 1(f) and 1(g)). The classifier based on the cytotoxic score exhibited favorable classification performance (Figure 1(h)); thus, the C0 cluster was regarded as the cytotoxicity-related cluster.

### 3.2. The Cytotoxic Classification in TCGA Cohort and KEGG Enrichment Analysis of T Cells

**Figure 2:**
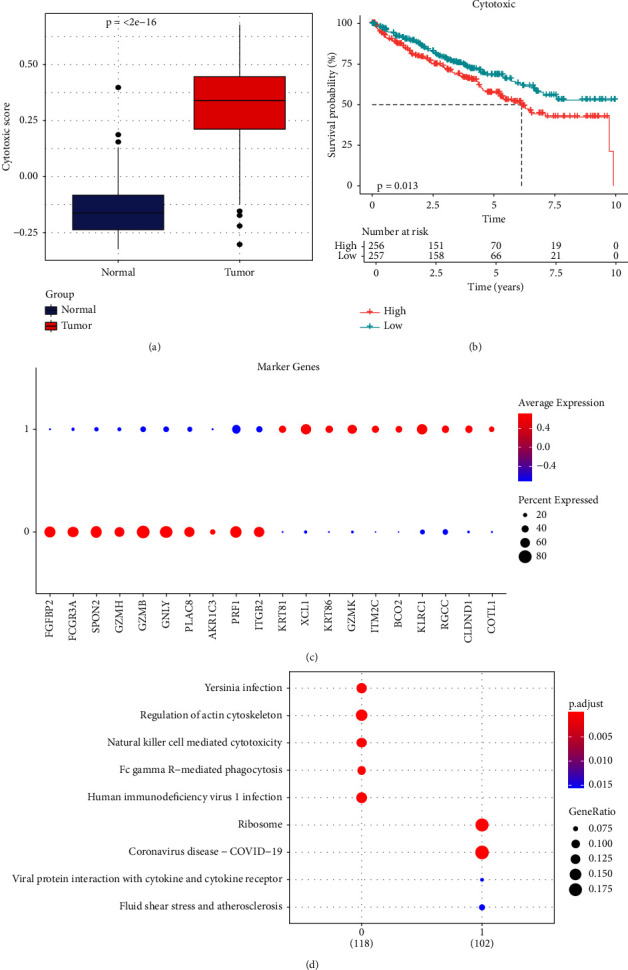
Cytotoxic classification in the TCGA cohort KEGG enrichment analysis of T cells. (a) The difference in cytotoxic scores between the tumor and adjacent normal tissues in the TCGA dataset. (b) Kaplan–Meier survival analysis of the low-cytotoxic score group versus the high-cytotoxic score group. (c) The bubble plot of the expression of top 10 maker genes in two T-cell subsets. (d) KEGG enrichment of marker genes in two T-cell subsets.

In addition, we calculated the cytotoxic score in the TCGA cohort and found that the tumor had a higher cytotoxic score via the Wilcoxon rank sum test (Figure 2(a)). The samples were further divided into high- and low-score groups through the median cytotoxic score, and the KM survival analysis showed that the low-score groups had better prognosis (Figure 2(b)). The bubble plot presented the top 10 differential marker genes in two T-cell clusters, and the antibody proteins such as FGFBP2, FCGR3A, SPON2, GZMH/B, GNLY, PLAC8, AKR1C3, PRF1, and ITGB2 were highly expressed in the C0 cluster (Figure 2(c)). The KEGG analysis suggested that the cytotoxicity is closely related to antitumor because from the results, we observed that the C0 cluster was closely associated with the Fc gamma R-mediated phagocytosis pathway, regulation of actin cytoskeleton, Yersinia infection, and natural killer cell-mediated cytotoxicity (Figure 2(d)).

### 3.3. Identification of Cytotoxicity-Related Molecular Subtypes in TCGA-KIRC Cohort

**Figure 3:**
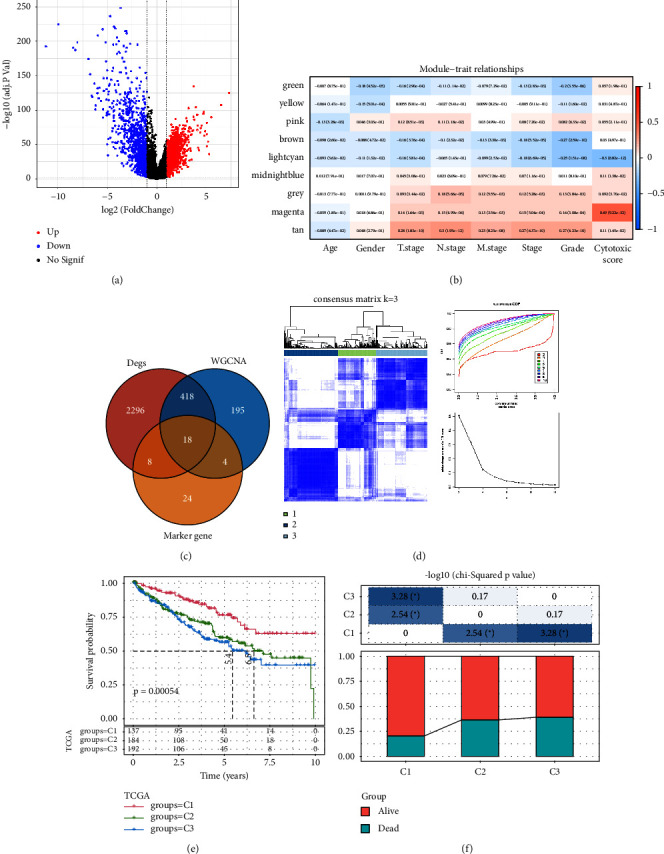
Identification of cytotoxicity-related molecular subtypes. (a) Volcanic plot of difference analysis between the tumor and paracancer normal samples in the TCGA dataset. (b) The correlation heatmap between the WGCNA-identified cytotoxicity-related module and different clinical features (each square indicated the corresponding correlation coefficient and *p* value). (c) The Venn plot of the identification of core cytotoxicity-related genes. (d) The clustering heatmap of samples and the CDF curve when consensus *k* = 3. (e) Kaplan–Meier survival analysis of three subtypes (C1 = cluster 1, C2 = cluster 2, and C3 = cluster 3). (f) Survival state difference of three subtypes in the TCGA dataset.

Difference analysis between ccRCC samples and paracancer normal samples in the TCGA-KIRC cohort obtained 2740 DEGs (Figure 3(a)). The WGCNA was used to identify the cytotoxicity-related genes. Hierarchical clustering (minModuleSize = 50, soft threshold *β* = 12, and distance >0.2) generated nine coexpression modules (Figure S2B), among which the grey module could not aggregate into other modules and was considered an ineffective module (Figures S2B and S2C). A significant positive relation (*R* = 0.49, *p* < 0.01) between the magenta modules and the cytotoxic score was detected (Figure 3(b)). The intersection of DEGs and magenta module genes and cluster 0 resulted in 18 overlapping genes (Figure 3(c)). To further identify the subtypes, the consensus clustering analysis was performed on ccRCC samples from TCGA based on the expression profiles of 18 overlapping genes. From the results of the CDF Delta area, it has a relatively stable clustering effect when the clustering is 3. Therefore, considering that the optimal clustering number (*k* value) of 3 is a better choice, we categorized the cohort into three (C1, C2, and C3) clusters (Figure 3(d)). The KM survival analysis of the three subtypes demonstrated that the C1 cluster had the best prognosis, while C3 had the worst prognosis (Figure 3(e)), and the survival state in the C1 cluster was significantly higher than in other clusters (Figure 3(f)).

### 3.4. Characterization of the TME in Different Subtypes

**Figure 4:**
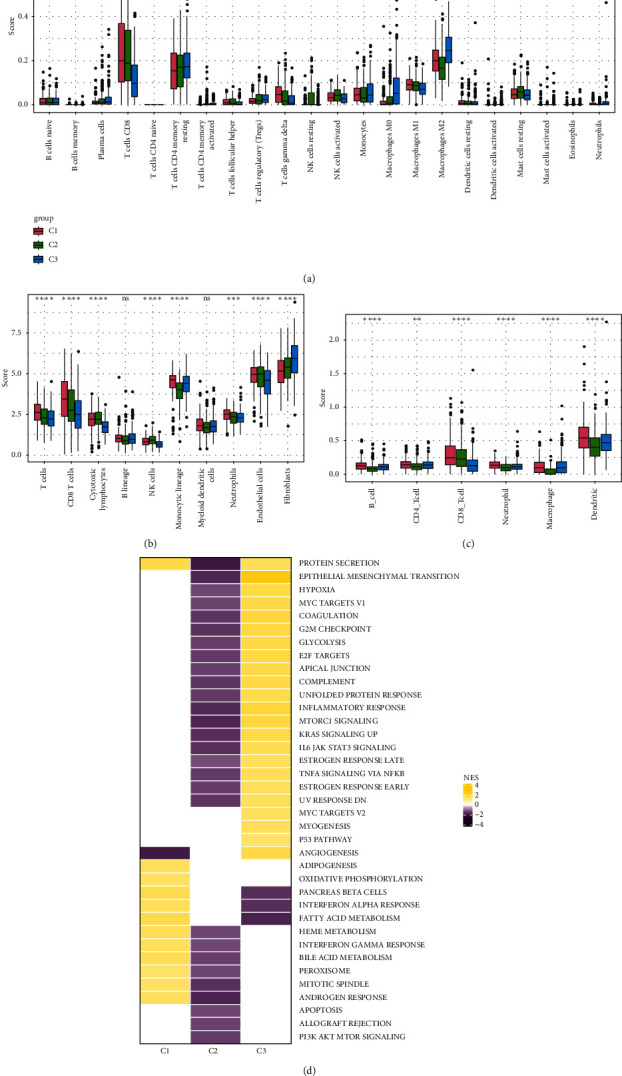
Characteristics of the immune microenvironment between subtypes. (a) The box plot of CIBERSORT immune infiltration in three subtypes. (b) MCP-counter immune infiltration in three subtypes. (c) TIMER immune infiltration in three subtypes. (d) The heatmap of differential activation pathways in three subtypes. Normalized Enrichment Score (NES), a measure of how enriched a particular set of genes is in a sample classification. ^∗^*p* < 0.05, ^∗∗^*p* < 0.01, ^∗∗∗^*p* < 0.001, ^∗∗∗∗^*p* < 0.0001, and ns represents no significant difference.

To explore the tumor microenvironment (TME) of the three molecular subtypes, we evaluated the relative abundance of 22 types of immune cells using the CIBERSORT, and the MCP-counter and the TIMER method were used to evaluate immune cell infiltration. We observed that the antitumor immune cells including T-cell gamma delta, macrophage M1, and T-cell CD8 are mainly contributing to the TME score and better prognosis in the C1 cluster (Figure 4(a)), which also had a higher immune cell infiltration score than patients in C2 and C3 groups (Figures 4(b) and 4(c)). The Pathway enrichment analysis uncovered that the metabolism-related pathways in the C1 group, and the immune, cell cycle, and some tumor-related pathways in the C3 group were activated, and most of the pathways in the C2 group were suppressed (Figure 4(d)). Taken together, the C1 group was characterized by the best prognosis, higher survival rate, and higher immune cell infiltration, and the C3 group was characterized by the worst prognosis.

### 3.5. Establishment of Cytotoxicity-Related Risk Model

**Figure 5:**
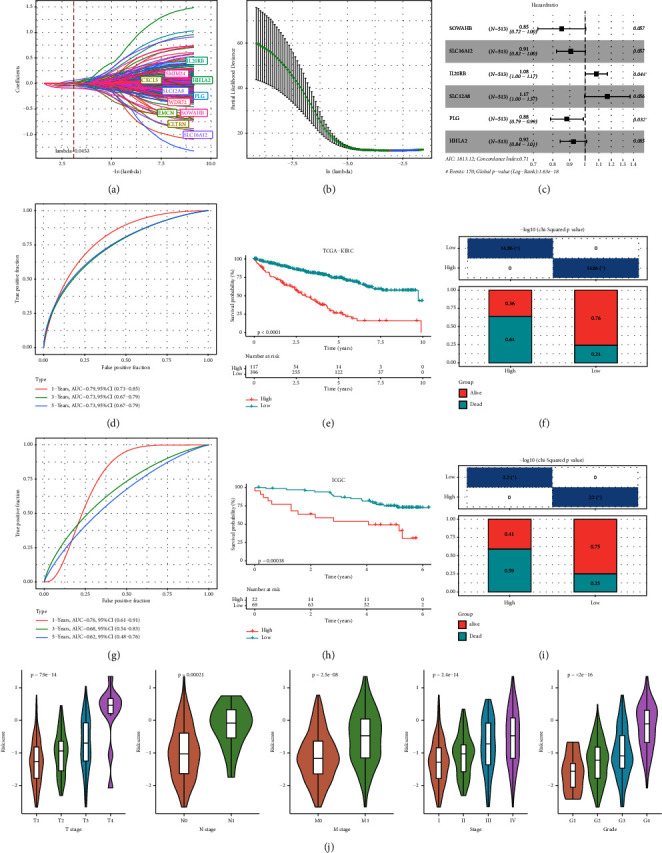
Establishment and validation of cytotoxicity-related risk model. (a) The trajectory of each independent variable as lambda changes. (b) The lambda confidence interval analysis. (c) Multifactor forest map of model genes. (d and e) The ROC curve and Kaplan–Meier curve of risk model in TCGA datasets. (f) Survival state differences between risk groups in TCGA datasets. (g and h) The ROC curve and Kaplan–Meier curve of risk model in ICGC datasets. (i) Survival state differences between risk groups in ICGC datasets. (j) RiskScore difference among different clinical grades in TCGA datasets.

The DEGs between the three subtypes were calculated by the limma R package. Finally, 81, 15, and 166 DEGs were identified in the C1, C2, and C3 groups, respectively, and after merging and deduplicating, 193 DEGs were obtained for further analysis. We conducted the univariate Cox analysis of the above 193 DEGs and identified 162 genes with greater prognostic influence (*p* < 0.05). The Lasso regression showing the trajectory of each independent variable revealed a mutual increase in the lambda gradually and the number of independent variable coefficients close to 0 (Figure 5(a)). The 10-fold cross-verification modeling and the lambda confidence interval analysis showed that the model was optimized when lambda (*λ*) = 0.0453 (Figure 5(b)), so the 11 target genes at *λ* = 0.0453 were selected for further study. Next, the stepwise multivariate regression analysis using the step Akaike information criterion (AIC) method in the MASS package was performed to optimize the model. Based on the hazard ratio, six genes were identified as key genes affecting prognosis (Figure 5(c)). The final model formula was as follows: RiskScore=(−0.161∗SOWAHB)+(−0.098∗SLC16A12)+0.079∗IL20RB+0.154∗SLC12A8+(−0.125∗PLG)+(−0.08∗HHLA2)). Among these genes, SOWAHB, SLC16A12, PLG, and HHLA2 had negative coefficients in the model, indicating that upregulating of their expression levels can improve the survival time of ccRCC patients.

### 3.6. Validation of Model Prediction Performance

According to the above RiskScore, high-risk and low-risk patients in TCGA-KIRC were grouped by the optimal cutoff point. The receiver operating characteristic (ROC) analysis revealed a high accuracy of the RiskScore to predict the long-term prognosis of ccRCC (AUC = 0.79, 0.73, and 0.73 at 1, 3, and 5 years, respectively) (Figure 5(d)). Patients with a high RiskScore tended to show the worst survival rate, as shown by the KM curves, with a 5-year survival of 21% (Figures 5(e) and 5(f)). The prognosis accuracy of the RiskScore was also excellent (AUC = 0.76, 0.68, and 0.62 at 1, 3, and 5 years, respectively) (Figure 5(g)), with the high-risk group showing a significantly lower 5-year survival of 47% (Figures 5(h) and 5(i)) in the validation set of ICGC. A comparison of the differences between the RiskScore and clinical grades shows that a higher RiskScore was associated with a higher clinical grade (Figure 5(j)). As a result, an effective six-gene signature to assess ccRCC prognosis was successfully created.

### 3.7. Screening Independent Risk Factors and Development of a Nomogram

**Figure 6:**
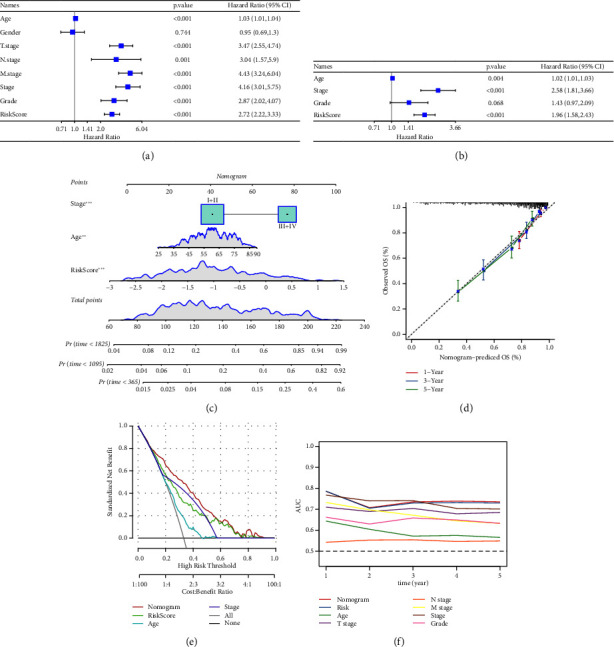
Identification of the independent risk factor and a nomogram development. (a) The forest plot of univariate Cox regression analysis between the RiskScore and clinical features. (b) The forest plot of the multivariate Cox analysis between the RiskScore and clinical features. (c) Nomogram model integrating the RiskScore, age, and stage. (d) Calibration plots for 1, 3, and 5 years of the nomogram. (e) The decision curve of a nomogram. (f) The timeROC curves with AUC of a variety of clinical features for overall survival (OS) at 1–5 years.

Univariate and multivariate Cox regression analyses were conducted to assess the efficacy of the RiskScore as an independent prognostic predictor by combining age, gender, and stage. We found that the RiskScore, age, and stage were independent predictors for ccRCC prognosis in TCGA patients (*p* < 0.01, HR = 2.72 (95% CI, 2.22–3.33),*p* < 0.01, HR = 1.03 (95% CI, 1.01–1.04), and *p* < 0.01, HR = 4.16 (95% CI, 3.01–5.75), Figure 6(a)). The multivariate Cox regression also demonstrated that the RiskScore was an independent predictor for the prognosis in ccRCC patients in the TCGA cohort (*p* < 0.01, HR = 1.96 (95% CI, 1.58–2.43), Figure 6(b)). A nomogram combining RiskScore, staging, and age was constructed, which had a wide range of RiskScore scores and contributed the most to the total score and thus had the greatest impact on survival prediction (Figure 6(c)) (*p* < 0.01). The calibration curve of 1, 3, and 5 years was close to the standard curve (Figure 6(d)), indicating that the nomogram can effectively predict the actual survival outcomes. The DCA analysis showed that the net benefit of the nomogram and RiskScore was significantly higher than the extreme curves, suggesting that the model had a better reliability (Figure 6(e)). The timeROC analysis revealed a higher AUC of the nomogram and RiskScore than other clinical indicators (Figure 6(f)), which suggested that the nomogram and RiskScore had the strongest prediction ability.

### 3.8. Immune Characteristics between Risk Groups

**Figure 7:**
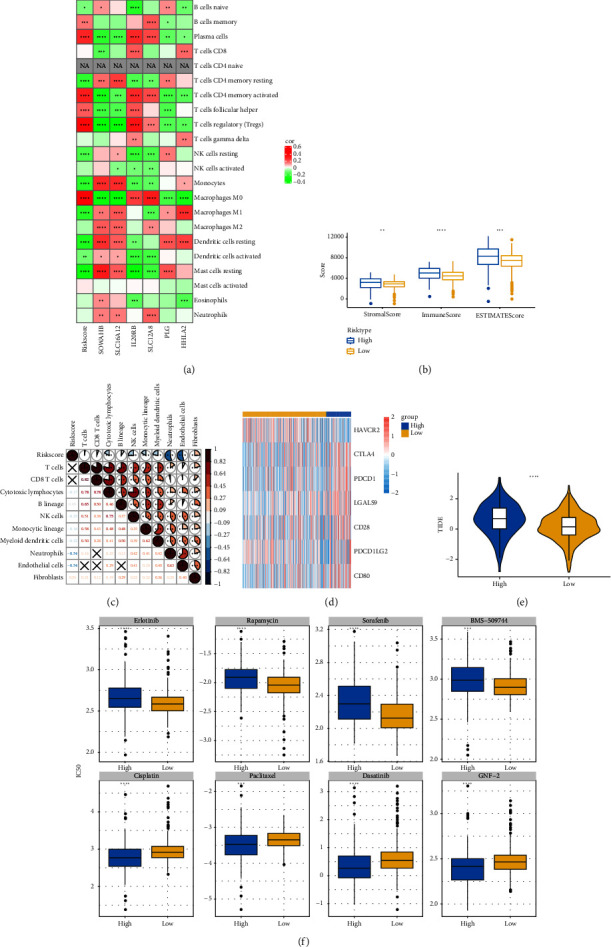
Immune characteristics between high-risk and low-risk groups. (a) Correlation between the RiskScore and CIBERSORT immune infiltration. (b) Difference in the estimated immune infiltration score between different risk groups. (c) Correlation between the RiskScore and MCP-counter immune infiltration. (d) Expression differences of common immune checkpoint genes between different risk groups. (e) TIDE score between different risk groups. (f) The drug sensitivity of patients in different risk groups. ^∗^*p* < 0.05, ^∗∗^*p* < 0.01, ^∗∗∗^*p* < 0.001, and ^∗∗∗∗^*p* < 0.0001.

The RiskScore was significantly positively (*p* < 0.05) correlated with the macrophage M0, T-cell CD4 memory activated, plasma cells, and T-cell regulatory (Tregs) and significantly negatively (*p* < 0.05) correlated with the dendritic and mast cell resting, monocytes, T-cell CD4 memory, NK cell resting, and macrophage M1, suggesting that the higher the RiskScore score, the weaker the immune-killing ability of the body. This applies to the IL20RB and PLG as well, and downregulation of these genes can lead to a low RiskScore (Figure 7(a)). The Analysis of the infiltration of immune cells using ESTIMATE showed higher stromal scores, immune scores, and ESTIMATEScore of the high-risk group (Figure 7(b)). The correlation between the RiskScore and immune infiltration of MCP-counter also confirmed that the RiskScore was significantly negatively linked with the infiltration of most immune cells (Figure 7(c)), suggesting that patients with a high RiskScore were less immune to tumors. Subsequently, we found that seven immune checkpoint genes showed a high expression in the high-risk group (Figure 7(d)). However, the potential clinical immunotherapy response analysis showed a higher TIDE score in high-risk patients (Figure 7(e)), suggesting a greater possibility of immune escape and relatively limited benefit from immunotherapy. In addition, the analysis of drug sensitivity showed a higher sensitivity in the low-risk group to BMS-509744, erlotinib, rapamycin, and sorafenib, while patients in the high-risk group were more sensitive to dasatinib, cisplatin, paclitaxel, and GNF-2 (Figure 7(f)). These results may provide guidance for the drug selection for the treatment of ccRCC.

### 3.9. Analysis of Pathway and Mutation Feature between Risk Groups

**Figure 8:**
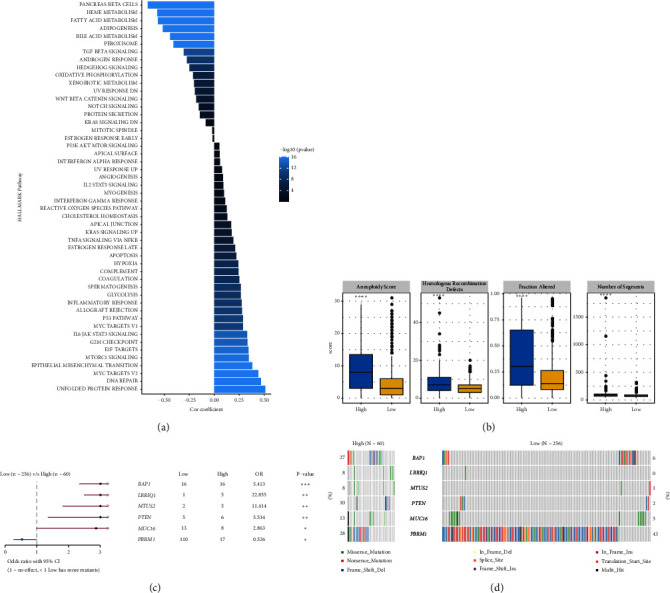
Analysis of pathway difference and mutations between risk groups. (a) Correlation between the RiskScore and HALLMARK pathway of the ssGSEA score. (b) Molecular characterization scores between different risk groups. (c) Differentially mutated genes between different risk groups. (d) Gene waterfall map of differential mutations between different risk groups. ^∗∗∗∗^*p* < 0.0001.

The correlation analysis between the pathway of the ssGSEA score and the RiskScore demonstrated a positive relation of the RiskScore to cell cycle-related pathways and a negative relation to metabolism-related pathways (Figure 8(a)). In addition, based on a previous pan-cancer study to characterize the mutation, the results showed that the high-risk groups were closely associated with the fraction altered, aneuploidy score, number of segments, and homologous recombination defects (Figure 8(b)). The Fisher analysis of gene mutation characteristics in the mutect2-treated dataset revealed that the PBRM1 is a highly mutated gene that affected tumorigenesis in the two groups, the BAP1 was a high-frequency mutation gene in the high-risk group (Figures 8(c) and 8(d)), and their function should be further studied.

## 4. Discussion

Renal cancer remains a serious urinary system problem with high mortality and morbidity [47]. The tumor microenvironment of ccRCC is usually accompanied by a high level of CD8+ T-cell infiltration [25], which is closely associated with patients' prognosis and immunotherapy efficacy in ccRCC. To enhance the understanding of biological functions of CD8+ T cell in TME, this study developed a cytotoxicity-related signature to provide therapeutic guidance and prognostic prediction for ccRCC patients. In this study, we identified T-cell subtypes based on single-cell sequencing data and used WGCNA to identify the gene modules most associated with cytotoxicity. In particular, in the TCGA dataset, the tumor tissues had a high-cytotoxic score and inferior prognosis, suggesting that the cytotoxic characteristics of TME are closely correlated with tumorigenesis.

Omics analysis has facilitated the discovery of disease markers [48]. In addition paper, using transcriptomics, we constructed a cytotoxicity-related prognostic prediction risk model consisting of six key genes (SOWAHB, SLC16A12, IL20RB, SLC12A8, PLG, and HHLA2), precise and concise, showing a preferable application prospect that benefits the prognostic evaluation personalized treatment for ccRCC patients. The SOWAHB, SLC16A12, PLG, and HHLA2 are considered protective factors, and the IL20RB, SLC12A8 are risk factors for ccRCC patients. The SOWAHB was reported as a potential regulator in ccRCC progression, but its functionality needs further verification. SLC16A12 is an identified creatine transporter [49], the lack of SLC16A12 usually leads to the low levels of creatine in chronic renal failure [50], and the comparatively lower expression of SLC16A12 in tumors often correlates with the worst prognosis [51]. This implies that SLC16A12 may exert its antitumor effects in ccRCC by maintaining the stability of energy metabolism in renal cells. In addition, the PLG is an antitumorigenic factor, and its hydrolysate contains angiotensin that will function against cancer progression [52]. The expression of PLG in ccRCC was lower than that in the adjacent normal tissue, indicating low levels of PLG in favor of ccRCC progression, leading to the worst overall survival (OS) [53]. The HHLA2 protein is expressed on antigen-presenting cells, and elevated HHLA2 is thought to be associated with a more severe pathology and poor prognosis in cancer patients [54–57], whereas several studies showed that patients with higher HHLA2 expression had better survival rate [58, 59]; this paradox can be explained, in part, by the dual role of HHLA2 in immunity and by the fact that the HHLA2 acts as a protective factor in this article. The high expressions of IL20RB and SLC12A8 usually predict the worst survival and were unfavorable prognostic biomarkers for ccRCC [60, 61]. This implies that all of these key genes have important roles in promoting the development of ccRCC, and their associated mechanisms of action still need to be explored in depth in future studies.

To evaluate the difference in the TME in the high- and low-risk groups, we performed the immune-infiltration landscape analyses. The high-risk group exhibited elevated immune infiltrations, including the stroma and immune score, which are associated with the worst prognosis [62, 63]. Meanwhile, the RiskScore was positively correlated with the immunosuppressive cells (M0 dormant macrophage and T-cell regulatory) and negatively correlated with the immune-activated cells (macrophage M1 and neutrophils), which explained an unfavorable ccRCC prognosis in patients showing high immune cell infiltration [25, 64]. The immunoediting theory suggests that the lack of immune cells, the presence of immunosuppressive cells, and high levels of immunosuppressive cytokines and fibrosis in TME might contribute to the immune escape of tumors [65–67]. The high-risk group had more infiltrations of immune cell and stroma cell as well as the high expression of immune checkpoint genes and a high TIME score, indicating the complex immune escape mechanism in the high-risk group. In the HALLMARK pathway analysis, the RiskScore was positively correlated with cell cycle-related pathways that can help to find more cell cycle check points. In addition, the high-risk group exhibited more variety of chromosome mutations and high mutant frequency of tumor suppressor BAP1 [68], while knockdown of BAP1 inhibited tumorigenicity and lung metastasis [69]; thus, the role of BAP mutations in the development of renal cancer needs to be further elucidated. PBMR1 is a hub gene that had higher mutation frequency in high- and low-risk groups. Usually, unbalanced PBMR1 leads to a shift from immune activation to immune suppression and is deregulated in tumors [70]; this could be a target gene for ccRCC patients.

Finally, there are some problems and limitations in this paper. First, our study was based only on samples from public databases due to the limited sample size; for this reason, future studies will incorporate more samples, including patients of different ethnicities and geographic regions, in order to improve the model's generalization ability and applicability. In addition, the specific functions and substrates of our screened key genes in ccRCC have not yet been thoroughly validated. Therefore, further in vivo and in vitro experiments, including cellular and animal models, are necessary to investigate the mechanism of action of the key genes. Finally, inferring partial immune characteristics from gene expression data alone may fail to fully resolve the full complexity and dynamics of the immune microenvironment. In the future, we will combine single-cell multiomics technology and spatial transcriptomics to deeply analyze the complex changes in the immune microenvironment of ccRCC.

## 5. Conclusion

Cytotoxicity-related prognosis genes were selected by performing univariate/multivariate and Lasso-Cox regression analysis and further used to build a risk model for accurately predicting the immunotherapy response and clinical outcomes of patients suffering from ccRCC. High- and low-risk ccRCC patients with different clinical features and immunogenomic landscapes were grouped by the gene classifier (RiskScore model), which can provide therapeutic guidance for ccRCC patients and improve the current individuated treatment options.

## Data Availability

The datasets generated during and analyzed during the current study are available from the corresponding author on reasonable request.

## Abbreviations

ccRCC: Clear cell renal cell carcinoma
TME: Tumor microenvironment
RCC: Renal cell carcinoma
TCGA: The Cancer Genome Atlas
ICGC: International Cancer Genome Consortium
KIRC: Kidney Renal Clear Cell Carcinoma
GEO: Gene Expression Omnibus
WGCNA: Weighted gene coexpression network analysis
KEGG: Kyoto Encyclopedia of Genes and Genomes
GSEA: Gene set enrichment analysis
DEGs: Differentially expressed genes
MAD: Medium absolute deviation
TIMER: Tumor Immune Estimation Resource
ROC: Receiver operating characteristic
AUC: Area under the curve
DCA: Decision curve analysis
CDF: Cumulative distribution function
TIDE: Tumor Immune Dysfunction and Exclusion
GSVA: Gene Set Variation Analysis.

## Supplementary Materials

Supplementary Materials Figure S1: single-cell dimension reduction clustering and T-cell recognition (Figure S1). (A) Violin diagram of the number of cells in the six samples before filtration. (B) Violin diagram of the number of cells filtered from the six samples. (C) Bars of cell numbers before and after filtration of the six samples. (D) Plot of tSNE_1 distribution of cells before batch treatment. (E) Plot of tSNE_1 distribution of cells after batch treatment. Figure S2: identification of molecular subtypes from the training set. (A) Soft threshold *β* of the scale-free network. (B, C) WGCNA filters' coexpression modules. (D) The number of genes in each module.

## References

[1] Zhang F., Wu P., Wang Y. (2020). Identification of significant genes with prognostic influence in clear cell renal cell carcinoma via bioinformatics analysis. *Translational Andrology and Urology*.

[2] Ljungberg B., Albiges L., Abu-Ghanem Y. (2022). European association of urology guidelines on renal cell carcinoma: the 2022 update. *European Urology*.

[3] Kotecha R. R., Motzer R. J., Voss M. H. (2019). Towards individualized therapy for metastatic renal cell carcinoma. *Nature Reviews Clinical Oncology*.

[4] Tian D., Shi Y., Lei L., Qiu X., Song T., Li Q. (2022). The transcriptional and immunological roles of Six2 in clear cell renal cell carcinoma. *Oncologie*.

[5] Zhou R., Li S., Xiao X. (2023). Aryl hydrocarbon receptor nuclear translocator 2 as a prognostic biomarker and immunotherapeutic indicator for clear cell renal cell carcinoma. *Biocell*.

[6] Yi P., Chongyuan H. (2023). PNO1 is a novel diagnostic and prognostic marker for clear cell renal cell carcinoma. *Journal of Biological Regulators & Homeostatic Agents*.

[7] Nengfeng Y., Gangfu Z., Congcong X. (2023). Identification of a prognostic risk-scoring model based on amino acid metabolism in renal clear cell carcinoma. *Journal of Biological Regulators & Homeostatic Agents*.

[8] Sun S., Xu L., Zhang X. (2021). Systematic assessment of transcriptomic biomarkers for immune checkpoint blockade response in cancer immunotherapy. *Cancers*.

[9] Zhou S., Lu Y., Chen Y., Gan W. (2023). Identification of an immunogenic cell death-related gene signature predicts survival and sensitivity to immunotherapy in clear cell renal carcinoma. *Scientific Reports*.

[10] Peng D., He A., He S. (2022). Ascorbic acid induced TET2 enzyme activation enhances cancer immunotherapy efficacy in renal cell carcinoma. *International Journal of Biological Sciences*.

[11] Gul A., Rini B. I. (2019). Adjuvant therapy in renal cell carcinoma. *Cancer*.

[12] Greten F. R., Grivennikov S. I. (2019). Inflammation and cancer: triggers, mechanisms, and consequences. *Immunity*.

[13] Vuong L., Kotecha R. R., Voss M. H., Hakimi A. A. (2019). Tumor microenvironment dynamics in clear-cell renal cell carcinoma. *Cancer Discovery*.

[14] Paijens S. T., Vledder A., de Bruyn M., Nijman H. W. (2021). Tumor-infiltrating lymphocytes in the immunotherapy era. *Cellular and Molecular Immunology*.

[15] Reese B., Silwal A., Daugherity E. (2020). Complement as prognostic biomarker and potential therapeutic target in renal cell carcinoma. *The Journal of Immunology*.

[16] Wu X., Jiang D., Liu H., Lu X., Lv D., Liang L. (2021). CD8(+) T cell-based molecular classification with heterogeneous immunogenomic landscapes and clinical significance of clear cell renal cell carcinoma. *Frontiers in Immunology*.

[17] Li Y., Wang Z., Jiang W. (2020). Tumor-infiltrating TNFRSF9(+) CD8(+) T cells define different subsets of clear cell renal cell carcinoma with prognosis and immunotherapeutic response. *OncoImmunology*.

[18] Dai S., Zeng H., Liu Z. (2021). Intratumoral CXCL13(+)CD8(+)T cell infiltration determines poor clinical outcomes and immunoevasive contexture in patients with clear cell renal cell carcinoma. *Journal for immunotherapy of cancer*.

[19] Stinchcombe J. C., Bossi G., Booth S., Griffiths G. M. (2001). The immunological synapse of CTL contains a secretory domain and membrane bridges. *Immunity*.

[20] Qu B., Pattu V., Junker C. (2011). Docking of lytic granules at the immunological synapse in human CTL requires Vti1b-dependent pairing with CD3 endosomes. *The Journal of Immunology*.

[21] Xu W., Atkins M. B., McDermott D. F. (2020). Checkpoint inhibitor immunotherapy in kidney cancer. *Nature Reviews Urology*.

[22] Thandra K. C., Barsouk A., Saginala K., Aluru J. S., Barsouk A. (2021). Epidemiology of lung cancer. *Contemporary Oncology*.

[23] Clark D. J., Dhanasekaran S. M., Petralia F. (2019). Integrated proteogenomic characterization of clear cell renal cell carcinoma. *Cell*.

[24] Zhang S., Zhang E., Long J. (2019). Immune infiltration in renal cell carcinoma. *Cancer Science*.

[25] Giraldo N. A., Becht E., Pagès F. (2015). Orchestration and prognostic significance of immune checkpoints in the microenvironment of primary and metastatic renal cell cancer. *Clinical Cancer Research*.

[26] Qi Y., Xia Y., Lin Z. (2020). Tumor-infiltrating CD39(+)CD8(+) T cells determine poor prognosis and immune evasion in clear cell renal cell carcinoma patients. *Cancer Immunology, Immunotherapy*.

[27] Satija R., Farrell J. A., Gennert D., Schier A. F., Regev A. (2015). Spatial reconstruction of single-cell gene expression data. *Nature Biotechnology*.

[28] Long Z., Sun C., Tang M. (2022). Single-cell multiomics analysis reveals regulatory programs in clear cell renal cell carcinoma. *Cell discovery*.

[29] Lu Y., Li K., Hu Y., Wang X. (2021). Expression of immune related genes and possible regulatory mechanisms in alzheimer’s disease. *Frontiers in Immunology*.

[30] Hänzelmann S., Castelo R., Guinney J. (2013). GSVA: gene set variation analysis for microarray and RNA-seq data. *BMC Bioinformatics*.

[31] Guo S., Wu J., Zhou W. (2021). Identification and analysis of key genes associated with acute myocardial infarction by integrated bioinformatics methods. *Medicine*.

[32] Langfelder P., Horvath S. (2008). WGCNA: an R package for weighted correlation network analysis. *BMC Bioinformatics*.

[33] Qiu C., Shi W., Wu H. (2021). Identification of molecular subtypes and a prognostic signature based on inflammation-related genes in colon adenocarcinoma. *Frontiers in Immunology*.

[34] Barakat A., Mittal A., Ricketts D., Rogers B. A. (2019). Understanding survival analysis: actuarial life tables and the Kaplan-Meier plot. *British Journal of Hospital Medicine*.

[35] Chen B., Khodadoust M. S., Liu C. L., Newman A. M., Alizadeh A. A. (2018). Profiling tumor infiltrating immune cells with CIBERSORT. *Methods in Molecular Biology*.

[36] Becht E., Giraldo N. A., Lacroix L. (2016). Estimating the population abundance of tissue-infiltrating immune and stromal cell populations using gene expression. *Genome Biology*.

[37] Li T., Fu J., Zeng Z. (2020). TIMER2.0 for analysis of tumor-infiltrating immune cells. *Nucleic Acids Research*.

[38] Yoshihara K., Shahmoradgoli M., Martínez E. (2013). Inferring tumour purity and stromal and immune cell admixture from expression data. *Nature Communications*.

[39] Zhang M., Zhu K., Pu H. (2019). An immune-related signature predicts survival in patients with lung adenocarcinoma. *Frontiers in Oncology*.

[40] Kassambara A., Kosinski M., Biecek P., Fabian S. (2017). Package “survminer” drawing survival curves using ‘ggplot2’. https://cran.r-project.org/web/packages/survminer/survminer.pdf.

[41] Zhang S., Tong Y. X., Zhang X. H. (2019). A novel and validated nomogram to predict overall survival for gastric neuroendocrine neoplasms. *Journal of Cancer*.

[42] Blanche P., Dartigues J. F., Jacqmin-Gadda H. (2013). Estimating and comparing time-dependent areas under receiver operating characteristic curves for censored event times with competing risks. *Statistics in Medicine*.

[43] Jiang P., Gu S., Pan D. (2018). Signatures of T cell dysfunction and exclusion predict cancer immunotherapy response. *Nature Medicine*.

[44] Geeleher P., Cox N., Huang R. S. (2014). pRRophetic: an R package for prediction of clinical chemotherapeutic response from tumor gene expression levels. *PLoS One*.

[45] Yang W., Soares J., Greninger P. (2012). Genomics of Drug Sensitivity in Cancer (GDSC): a resource for therapeutic biomarker discovery in cancer cells. *Nucleic Acids Research*.

[46] Thorsson V., Gibbs D. L., Brown S. D. (2018). The immune landscape of cancer. *Immunity*.

[47] Li C., Zhang T., Yan M. (2024). Exploring genes within the glutathione peroxidase family as potential predictors of prognosis in papillary renal cell carcinoma. *Oncologie*.

[48] Shahrajabian M. H., Sun W. (2023). Survey on multi-omics, and multi-omics data analysis, integration and application. *Current Pharmaceutical Analysis*.

[49] Takahashi M., Kishimoto H., Shirasaka Y., Inoue K. (2020). Functional characterization of monocarboxylate transporter 12 (SLC16A12/MCT12) as a facilitative creatine transporter. *Drug Metabolism and Pharmacokinetics*.

[50] Verouti S. N., Lambert D., Mathis D. (2021). Solute carrier SLC16A12 is critical for creatine and guanidinoacetate handling in the kidney. *American Journal of Physiology - Renal Physiology*.

[51] Cui H., Shan H., Miao M. Z. (2020). Identification of the key genes and pathways involved in the tumorigenesis and prognosis of kidney renal clear cell carcinoma. *Scientific Reports*.

[52] Cao Y., Xue L. (2004). Angiostatin. *Seminars in Thrombosis and Hemostasis*.

[53] Wang S., Yu Z. H., Chai K. Q. (2019). Identification of EGFR as a novel key gene in clear cell renal cell carcinoma (ccRCC) through bioinformatics analysis and meta-analysis. *BioMed Research International*.

[54] Jing C. Y., Fu Y. P., Yi Y. (2019). HHLA2 in intrahepatic cholangiocarcinoma: an immune checkpoint with prognostic significance and wider expression compared with PD-L1. *Journal for immunotherapy of cancer*.

[55] Chen D., Chen W., Xu Y. (2019). Upregulated immune checkpoint HHLA2 in clear cell renal cell carcinoma: a novel prognostic biomarker and potential therapeutic target. *Journal of Medical Genetics*.

[56] Wei L., Tang L., Chang H., Huo S., Li Y. (2020). HHLA2 overexpression is a novel biomarker of malignant status and poor prognosis in gastric cancer. *Human Cell*.

[57] Zhu Z., Dong W. (2018). Overexpression of HHLA2, a member of the B7 family, is associated with worse survival in human colorectal carcinoma. *OncoTargets and Therapy*.

[58] Boor P. P. C., Sideras K., Biermann K. (2020). HHLA2 is expressed in pancreatic and ampullary cancers and increased expression is associated with better post-surgical prognosis. *British Journal of Cancer*.

[59] Zhang Z., Liu J., Zhang C. (2020). Over-expression and prognostic significance of HHLA2, a new immune checkpoint molecule, in human clear cell renal cell carcinoma. *Frontiers in Cell and Developmental Biology*.

[60] Guo H., Jiang S., Sun H. (2022). Identification of IL20RB as a novel prognostic and therapeutic biomarker in clear cell renal cell carcinoma. *Disease Markers*.

[61] Zhang Q., Liu Y., Chen P. (2021). Solute carrier family 12 member 8 (SLC12A8) is a potential biomarker and related to tumor immune cell infiltration in bladder cancer. *Bioengineered*.

[62] Wang H., Wu X., Chen Y. (2019). Stromal-immune score-based gene signature: a prognosis stratification tool in gastric cancer. *Frontiers in Oncology*.

[63] Bremnes R. M., Dønnem T., Al-Saad S. (2011). The role of tumor stroma in cancer progression and prognosis: emphasis on carcinoma-associated fibroblasts and non-small cell lung cancer. *Journal of Thoracic Oncology*.

[64] He B., Zhao Z., Cai Q. (2020). miRNA-based biomarkers, therapies, and resistance in Cancer. *International Journal of Biological Sciences*.

[65] Xiao Y., Ma D., Zhao S. (2019). Multi-omics profiling reveals distinct microenvironment characterization and suggests immune escape mechanisms of triple-negative breast cancer. *Clinical Cancer Research*.

[66] Spranger S. (2016). Mechanisms of tumor escape in the context of the T-cell-inflamed and the non-T-cell-inflamed tumor microenvironment. *International Immunology*.

[67] Schreiber R. D., Old L. J., Smyth M. J. (2011). Cancer immunoediting: integrating immunity’s roles in cancer suppression and promotion. *Science (New York, N.Y.)*.

[68] Carbone M., Harbour J. W., Brugarolas J. (2020). Biological mechanisms and clinical significance of BAP1 mutations in human cancer. *Cancer Discovery*.

[69] Qin J., Zhou Z., Chen W. (2015). BAP1 promotes breast cancer cell proliferation and metastasis by deubiquitinating KLF5. *Nature Communications*.

[70] Mota S. T. S., Vecchi L., Zóia M. A. P. (2019). New insights into the role of polybromo-1 in prostate cancer. *International Journal of Molecular Sciences*.

