# Supplementary figures and images for: Identification and Validation of Cytotoxicity-Related Features to Predict Prognostic and Immunotherapy Response in Patients with Clear Cell Renal Cell Carcinoma
Source: Genet Res (Camb). 2024 Aug 30;2024:3468209. doi: 10.1155/2024/3468209 (PMC11379509; doi:10.1155/2024/3468209)

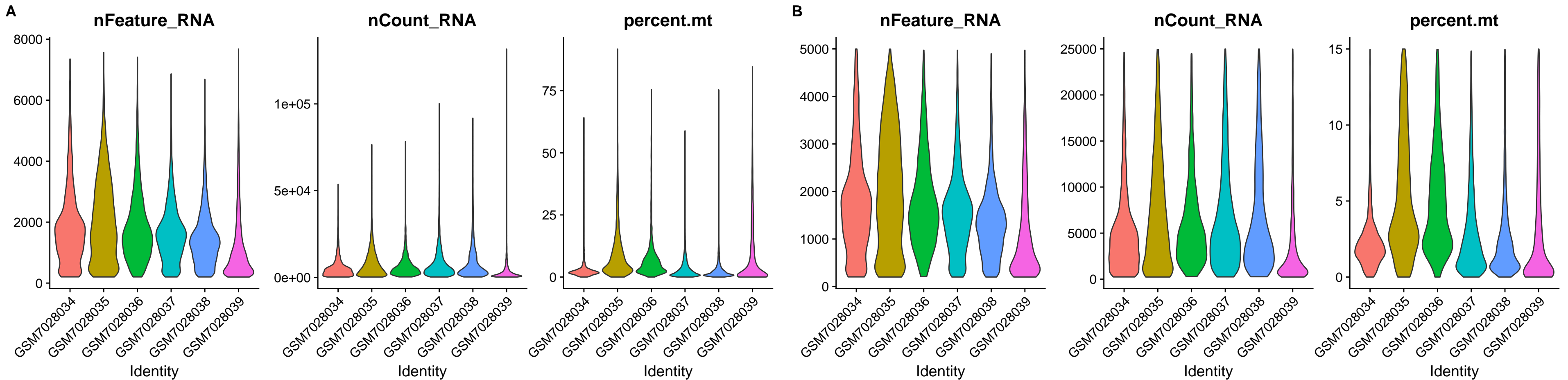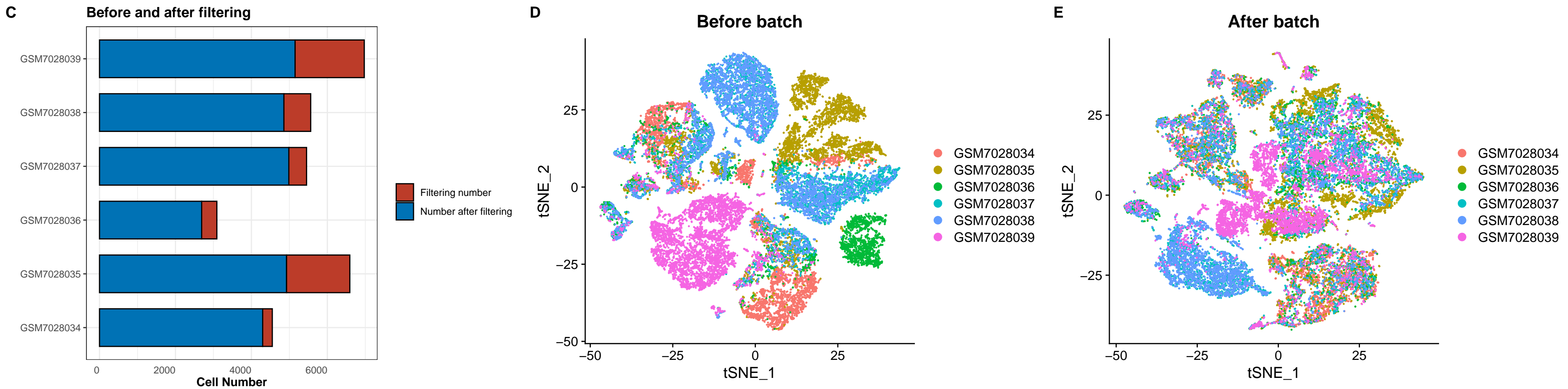

Supplement: Supplementary Materials — Figure S1: single-cell dimension reduction clustering and T-cell recognition (Figure S1). (A) Violin diagram of the number of cells in the six samples before filtration. (B) Violin diagram of the number of cells filtered from the six samples. (C) Bars of cell numbers before and after filtration of the six samples. (D) Plot of tSNE_1 distribution of cells before batch treatment. (E) Plot of tSNE_1 distribution of cells after batch treatment. Figure S2: identification of molecular subtypes from the training set. (A) Soft threshold β of the scale-free network. (B, C) WGCNA filters' coexpression modules. (D) The number of genes in each module. [file 3468209.f1.zip › FigS1.pdf]

**A**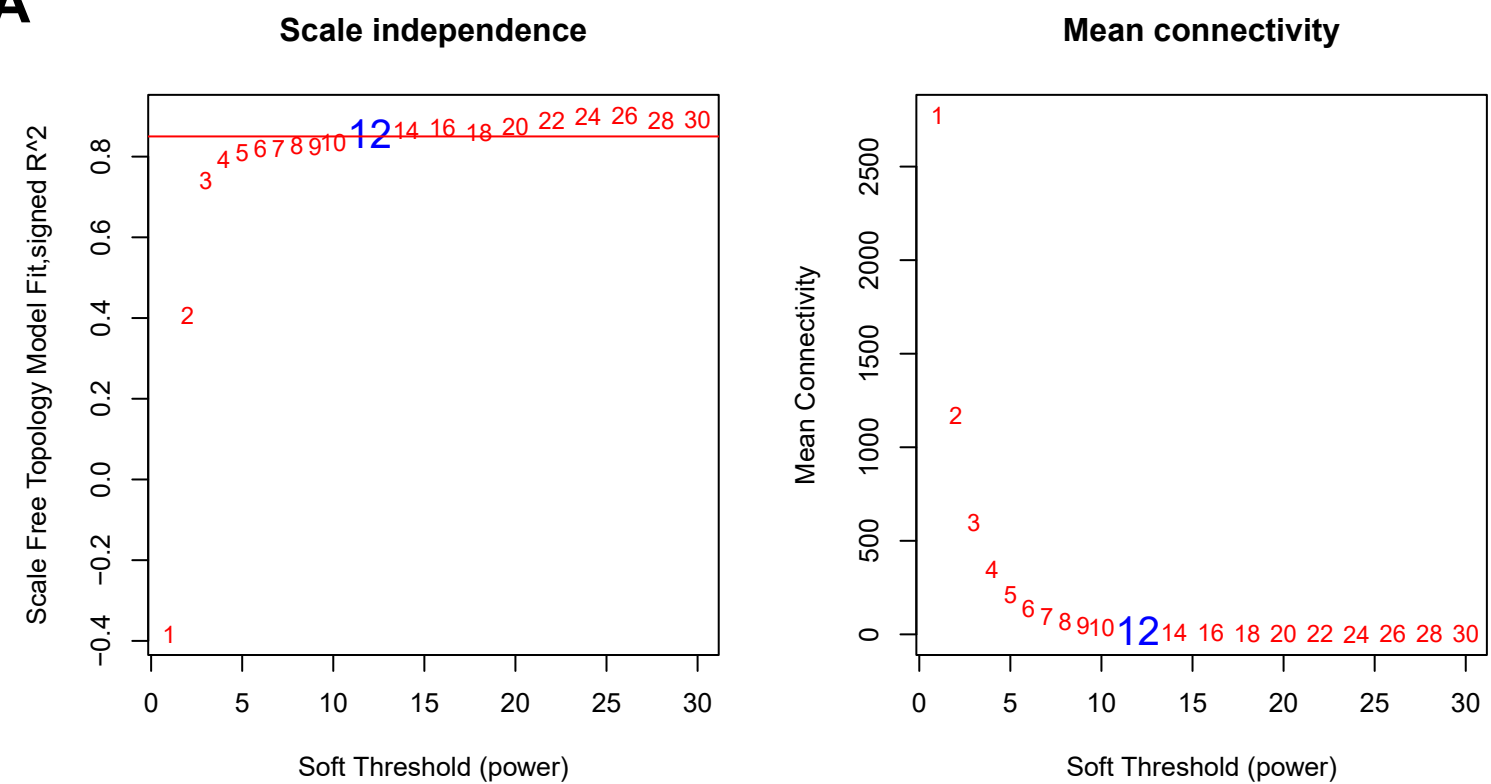**B**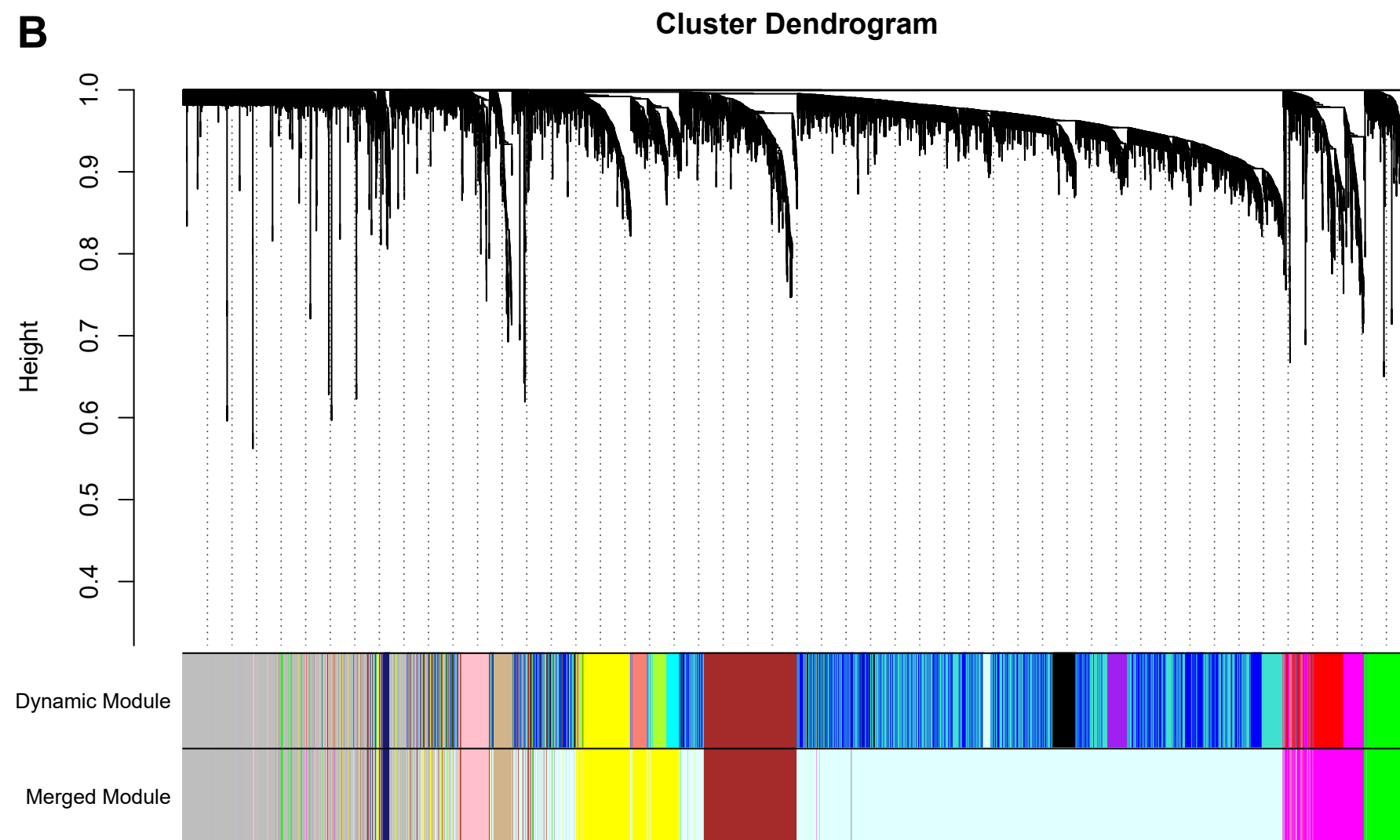**C**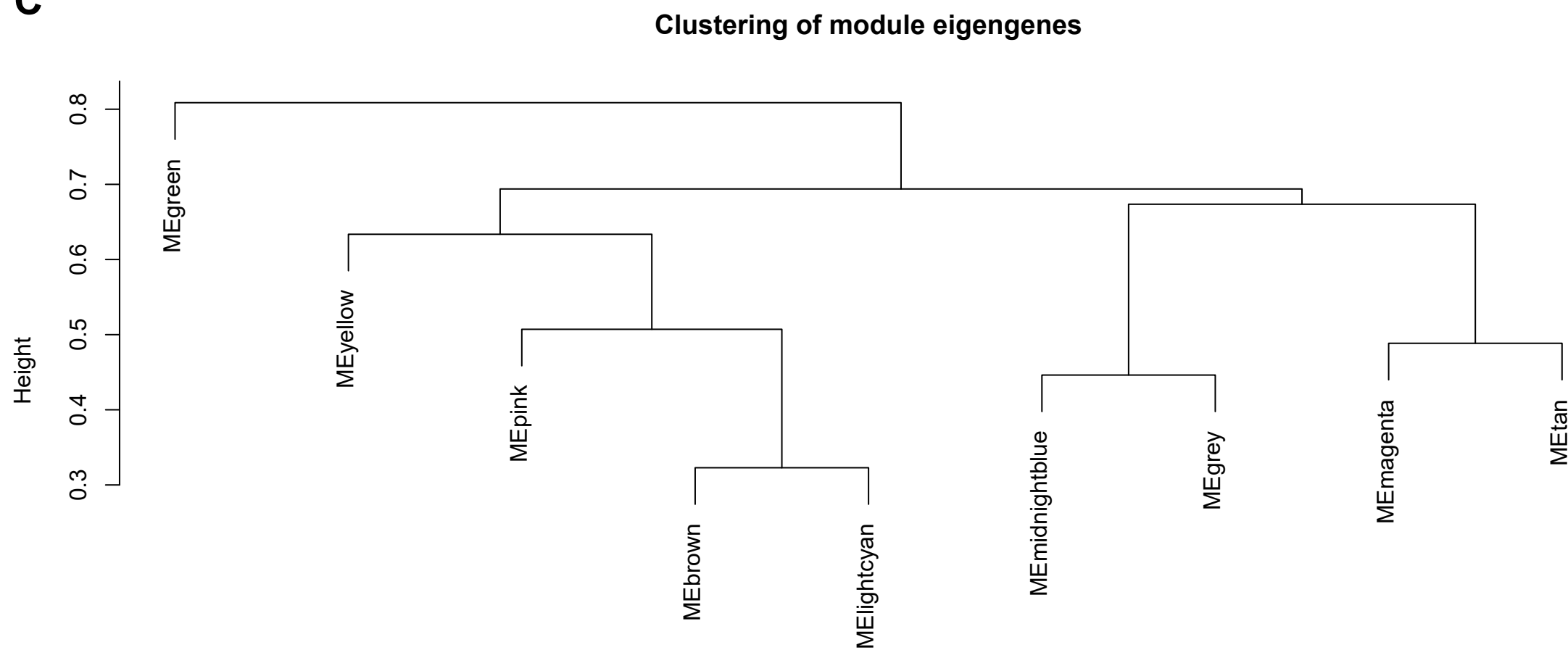**D**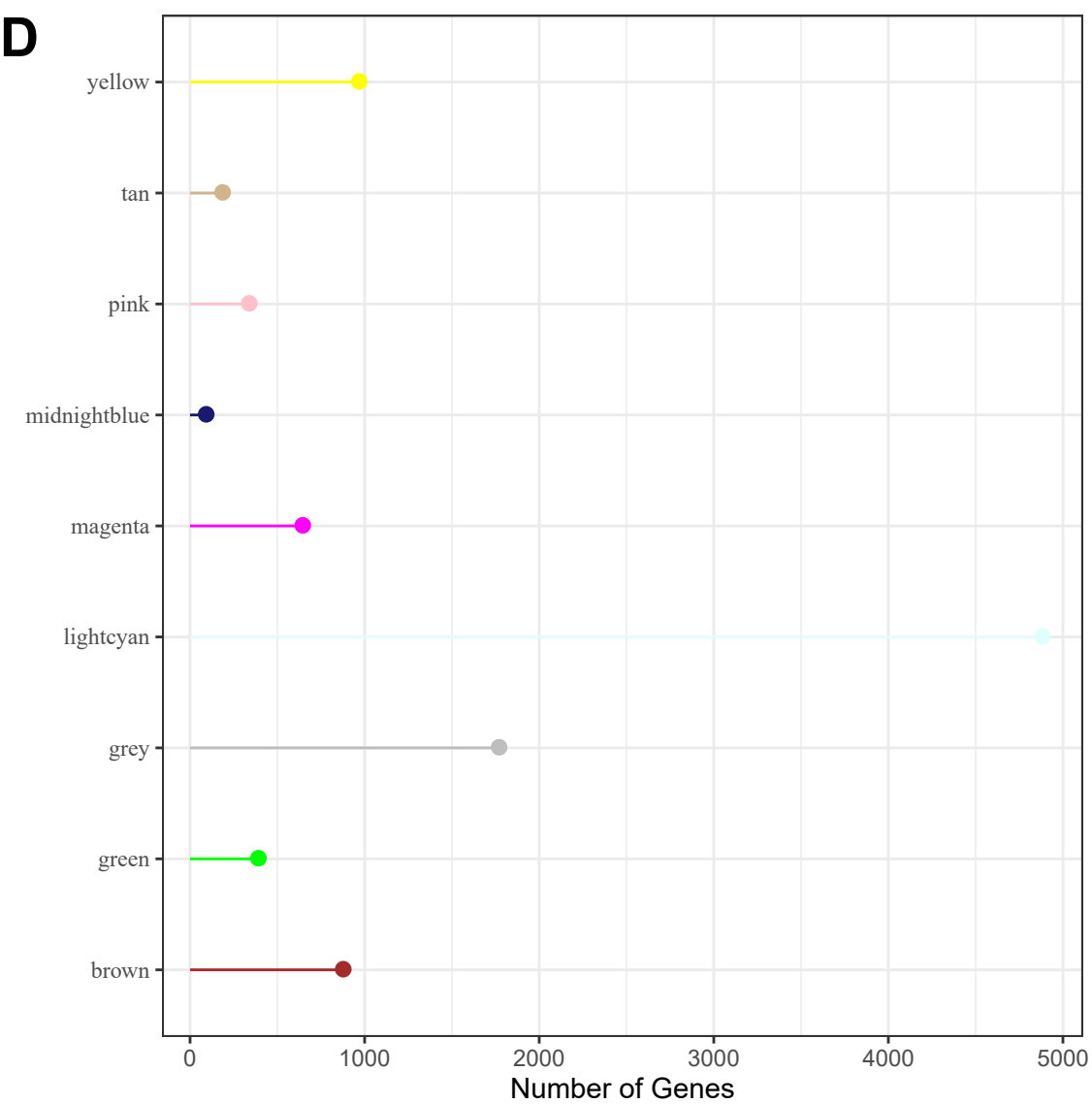

Supplement: Supplementary Materials — Figure S1: single-cell dimension reduction clustering and T-cell recognition (Figure S1). (A) Violin diagram of the number of cells in the six samples before filtration. (B) Violin diagram of the number of cells filtered from the six samples. (C) Bars of cell numbers before and after filtration of the six samples. (D) Plot of tSNE_1 distribution of cells before batch treatment. (E) Plot of tSNE_1 distribution of cells after batch treatment. Figure S2: identification of molecular subtypes from the training set. (A) Soft threshold β of the scale-free network. (B, C) WGCNA filters' coexpression modules. (D) The number of genes in each module. [file 3468209.f1.zip › FigS2.pdf]
